# CT-Based Radiomics Signature for the Preoperative Discrimination Between Head and Neck Squamous Cell Carcinoma Grades

**DOI:** 10.3389/fonc.2019.00821

**Published:** 2019-08-30

**Authors:** Wenli Wu, Junyong Ye, Qi Wang, Jin Luo, Shengsheng Xu

**Affiliations:** ^1^Department of Radiology, The First Affiliated Hospital, Chongqing Medical University, Chongqing, China; ^2^Key Laboratory of Optoelectronic Technology and Systems of the Ministry of Education, Chongqing University, Chongqing, China; ^3^Department of Information, The First Affiliated Hospital, Chongqing Medical University, Chongqing, China

**Keywords:** head and neck cancer, grade, computed tomography, radiomics signature, biomarker

## Abstract

**Background:** Radiomics has been widely used to non-invasively mine quantitative information from medical images and could potentially predict tumor phenotypes. Pathologic grade is considered a predictive prognostic factor for head and neck squamous cell carcinoma (HNSCC) patients. A preoperative histological assessment can be important in the clinical management of patients. We applied radiomics analysis to devise non-invasive biomarkers and accurately differentiate between well-differentiated (WD) and moderately differentiated (MD) and poorly differentiated (PD) HNSCC.

**Methods:** This study involved 206 consecutive HNSCC patients (training cohort: *n* = 137; testing cohort: *n* = 69). In total, we extracted 670 radiomics features from contrast-enhanced computed tomography (CT) images. Radiomics signatures were constructed with a kernel principal component analysis (KPCA), random forest classifier and a variance-threshold (VT) selection. The associations between the radiomics signatures and HNSCC histological grades were investigated. A clinical model and combined model were also constructed. Areas under the receiver operating characteristic curves (AUCs) were applied to evaluate the performances of the three models.

**Results:** In total, 670 features were selected by the KPCA and random forest methods from the CT images. The radiomics signatures had a good performance in discriminating between the two cohorts of HNSCC grades, with an AUC of 0.96 and an accuracy of 0.92. The specificity, accuracy, sensitivity, positive predictive value (PPV), and negative predictive value (NPV) of the abovementioned method with a VT selection for determining HNSCC grades were 0.83, 0.92, 0.96, 0.94, and 0.91, respectively; without VT, the corresponding results were 0.70, 0.83, 0.88, 0.80, and 0.84. The differences in accuracy, sensitivity and NPV were significant between these approaches (*p* < 0.05). The AUCs with VT and without VT were 0.96 and 0.89, respectively (*p* < 0.05). Compared to the combined model and the radiomics signatures, The clinical model had a worse performance, and the differences were significant (*p* < 0.05). The combined model had the best performance, but the difference between the combined model and the radiomics signature weren't significant (*p* > 0.05).

**Conclusions:** The CT-based radiomics signature could discriminate between WD and MD and PD HNSCC and might serve as a biomarker for preoperative grading.

## Introduction

Head and neck squamous cell carcinoma (HNSCC) is the sixth most common malignant tumor worldwide. Many factors affect the prognosis of patients with HNSCC; among these factors, the histological differentiation grade was reported to correlate with lymph node status, distant metastases, survival and prognosis (1–4). A pretreatment histopathologic grade evaluation for HNSCC provides information for clinical decision making. Although the histological differentiation grade is routinely confirmed by biopsy and surgical resection in many head and neck cancer centers, invasive biopsy is sometimes of little predictive value in early-stage oral SCC (5). In addition, intra-tumor heterogeneity is an issue. Biopsies do not exactly reflect the overall pathophysiology of the lesion.

Some non-invasive functional imaging modalities have been developed in the clinic, such as diffusion-weighted imaging (DWI), dynamic contrast-enhanced magnetic resonance imaging (DCE-MRI), perfusion-weighted imaging (PWI), and positron emission tomography (PET), all of which have been applied in the grading of HNSCC (6–8). These imaging modalities play important roles in the evaluation of disease grade to some extent, but combining clinical visual assessments is necessary to increase the overall accuracy.

Radiomics, which refers to an enhanced deep analysis of the molecular aspects of tumors and accounts for intrinsic susceptibility in the long-term follow-up, is a qualitative and quantitative analysis of a large amount of radiologic data extracted in a high-throughput manner to obtain predictive or prognostic information from cancer patients (9, 10). Radiomics is suitable for providing some predictive, classifying, and prognostic information for HNSCC patients (11–13). A few radiomics studies have been conducted based on MRI regarding the staging and grading of HNSCC (14–16). Although the vast majority of radiomics analyses were conducted on CT images, no studies exist about radiomics models based on CT signatures to differentiate HNSCC grades.

A large number of machine-learning methods were used to evaluate their applying values in HNSCC patients (17, 18). In this study, we will use another analysis method based on CT radiomics signatures to evaluate its predictive value in differentiating between HNSCC grades (WD vs. MD/PD).

## Materials and Methods

### Study Population

**Figure 1:**
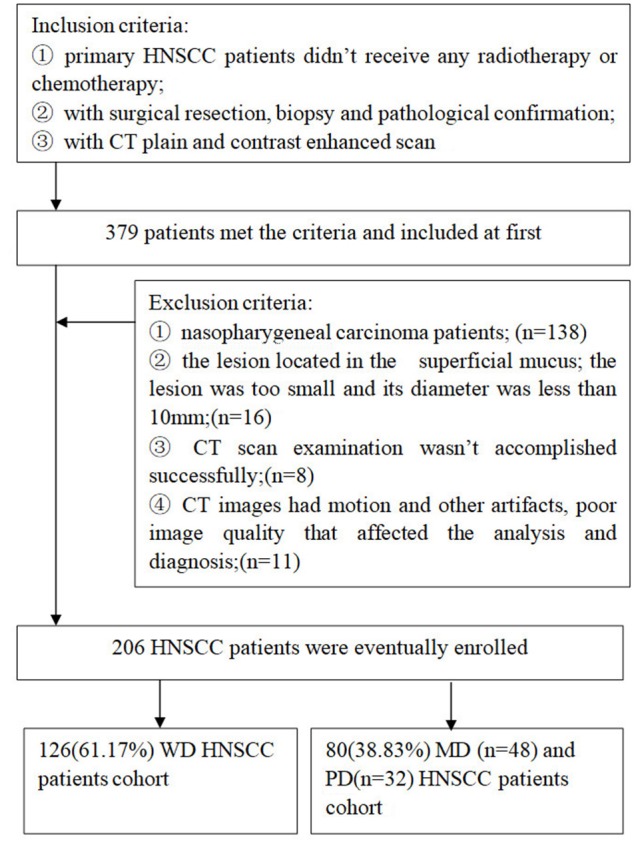
Flowchart showed patients selection for the study.

We collected patients with head and neck tumors confirmed to be SCC by surgical pathology in our hospital from January 2012 to February 2018. This study was approved by the institutional review board of our hospital (approval number 2019-178), and informed consent was waived. All patients underwent both precontrast and multiple-phase pretreatment contrast enhanced multi-slice spiral computed tomography (MSCT) scans. In this study, the patients were chosen and excluded according to the criteria presented in Figure 1. A total of 206 consecutive patients were identified met the criteria. These patients were randomly divided into a training cohort and a testing cohort at a ratio of 9:1 by a computer. We retrospectively analyzed the clinical information of all patients, including race, age, sex, tumor sites, tumor differentiation, tumor node metastasis (TNM) classification, and stage.

### CT Image Acquisition

All CT scans were performed using a GE Discovery 750 HD (GE Healthcare, Milwaukee, WI, USA) multidetector CT scanner. The CT scanning area was from the skull base down to the thorax inlet. The scanning parameters were as follows: 120 kV; 80 mA; pitch 0.984; detector collimation, 64 × 0.625 mm; rotation time, 0.6 s; matrix, 512 × 512; section thickness, 5 mm; and field of view, 220–250 × 220–250 mm. First, a non-contrast enhanced CT scan was performed, and then a contrast-enhanced CT scan was performed in the arterial phase (25–30 s), portal venous phase (60–65 s), and delayed phase (120 s), after an intravenous injection of non-ionic iodinated contrast medium (Ultravist 370, Bayer Schering Pharma, Berlin, Germany) (dose 1.5 mL/kg, injection rate 3.5 mL/s).

### Image Analysis

#### Preprocessing

##### Lesion segmentation and labeling

We segmented and labeled the lesions on picture archiving and communication systems (PACS) (Carestream Health Inc., Rochester, NY, USA). First, the doctor's terminal was opened to view the enrolled patients' CT images, especially the portal venous phase contrast-enhanced images, and then the slice on which the lesion was the most obviously displayed was determined. Second, the image window width was adjusted to 350 Hounsfield units (HU), and the window level was adjusted to 40 HU. Third, the curve measurement button on the toolbar was clicked to set the line color to red. Then, the largest solid part of the tumor was encircled to include the markedly enhanced area and excluded the necrotic or cystic areas. The enclosed area was recognized as a region of interest (ROI) and could be round, oval or another irregular shape. The area of the ROI was more than 1 cm^2^, which guaranteed a large enough area for analysis. Finally, the image with the ROI was exported and saved in a JPG format for subsequent processing. The segmentation and labeling processes were performed by two head and neck radiologists (J.F. and Y.T. with 5 and 10 years of diagnostic experience, respectively). Any discrepancies that occurred were resolved by a consensus between the two radiologists.

The goal of preprocessing was to delineate tumor regions, but first, the coordinates of the tumor area needed to be detected. Because the CT image was almost gray, the red line could easily be detected by a sliding a 64 × 64 rectangle to scan the whole image from the left top with step size of 1. This sliding rectangle recorded the coordinates of the vertex as soon as the rectangle came into contact with the closed red line.

We used a 64 × 64 window to scan the whole image with a step size of 1. Once the closed red line was found, the scanning process was stopped. Since we used the red line to contour the tumor, the window had a 100% overlap with the tumor at this time. The segmentation process was performed by two head and neck radiologists (W.X. and C.Y. with 8 and 11 years of diagnostic experience, respectively). We used an original non-annotated image in case the annotated red line interfered with the prediction. The coordinates recorded by the sliding rectangle could help delineate a 64 × 64 tumor region on the original image. These delineated images are called patches. Only the tumor region was considered when discriminating WD HNSCC from MD/PD HNSCC so that we could focus on the tumor and reduce the amount of noise interference. In addition, compared to a complete tumor region, a 64 × 64 patch contained some tissues around the tumor, which could also contribute to the tumor grade.

**Figure 2:**
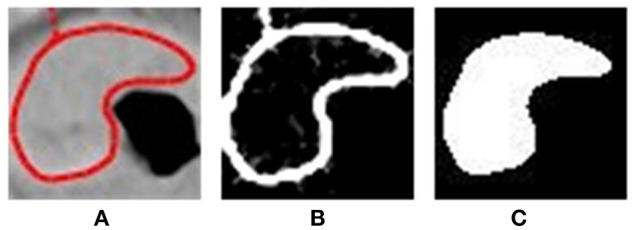
Steps of preprocessing: **(A)** cutting off the patches of ROI; **(B)** detecting the edge; **(C)** fulfilling the edge and generating mask.

To extract the shape features, we need an additional mask to describe the shape of the tumor. We extracted the edge of the patch, primarily by keeping only the red parts of the image and then filling in the edge and erasing the small annotated area to generate a mask. We used both the segmented patches and masks to extract all features (Figure 2).

##### Extracting radiomics features

**Figure 3:**
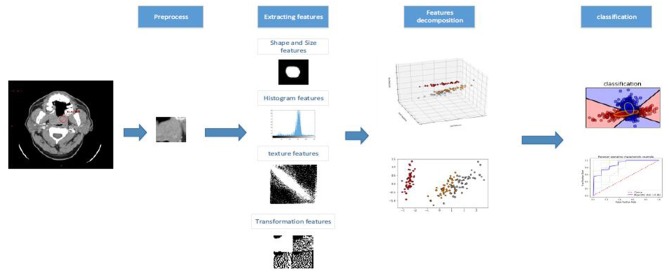
The workflow of proposed kernelized radiomics model in HNSCC.

We extracted 670 radiomics features from the portal venous phase contrast-enhanced CT images. These features quantified the phenotypic HNSCC characteristics and were divided into four feature groups: shape and size features; histogram features; texture features; and transformation features. All features are shown in Tables S1–S4, and we used all features to construct the random forest model. The workflow of the radiomics analysis is shown in Figure 3. The preprocessing and feature extraction methods were coded in MATLAB and python using scikit-image (19).

##### Feature decomposition and classification

Although 670 features were extracted for each patient, these features did not contribute equally to discriminating between WD HNSCC and MD/PD HNSCC. The features with low discrimination capabilities or those highly correlated with each other would overfit the classifiers and lead to a poor outcome. Therefore, feature decomposition was performed to find a set of candidate features with excellent discrimination capabilities and significant differences before grade prediction. In addition to feature selection, feature decomposition could also generate new features that are more capable of discrimination and have less correlation with each other than the original features. We used a non-linear kernelization method in the analysis. KPCA, which could be seen as a non-linear version of PCA, is a perfect answer to non-linear requests. In this paper, the following radial basis function (RBF) kernel was used:

(1) $$\begin{array}{l} k_{RBF}\left(x_{i},x_{j}\right)=exp\left(-\frac{||x_{i}-x_{j}\;|{|}^{2}}{2\sigma^{2}}\right). \end{array}$$

Then, the features extracted from the CT image could be processed by the KPCA algorithm with a RBF kernel. The decomposition and classification methods were implemented using scikit-learn (20), followed by a random forest classifier, and we finally obtained our proposed kernelized radiomics model. All experiments were performed under a Windows OS on a machine with CPU Intel Xeon E5 2687W V3, GPU NVIDIA GeForce 1080ti, and 16^*^8GB of RAM.

##### Kernelized radiomics model building

To build our kernelized radiomics model, we first decided on the dimensions of the kernelized features. When using the RBF kernel, we tuned the dimension value from 30 to 200 with steps of 10.

Because a little imbalance existed between the positive and negative samples in our dataset, AUC, instead of accuracy, was used to select the dimension value.

Since the dimension of the kernelized features had been decided, we still needed to select the classifier parameters. For an ensemble learning method using random feature selection, the main factors that could affect the performance of the random forest model are the number of basic learners (decision tree), maximum depth of each decision tree and number of randomly selected features. We used a gidsearch to search for the best values of these parameters, which tuned one parameter while freezing the others.

We removed features with a training set variance lower than 0.8. We used the python and sklearn library to implement this method, which first calculated the variance of each feature and then removed features with a low variance.

##### Clinical and combined model building

According to previous studies (1, 4, 21–25), some clinical and radiological characteristics are related to the differentiation grades of HNSCC. The TN classification, stage and enhancement types were selected as the clinical parameters for clinical model building (Supplementary Data Sheet 2). These clinical and radiological characteristics and radiomics signatures were integrated to build the combined model.

### Statistical Analysis

The discriminating performance of this model was evaluated with ROC curves and AUCs, and these values were compared using DeLong tests. The differences in clinical characteristics between the training and validation sets were evaluated using Student's *t*-tests and chi-square tests, and a *p* < 0.05 was considered statistically significant. IBM SPSS software ver. 24 (IBM Corp., Armonk, NY, USA) and open-source machine learning studio were used for statistical analysis. The inter-observer agreement in evaluating the enhancement types (homogeneous/heterogeneous) was assessed with kappa statistics: a kappa value between 0.00 and 0.20 indicates a slight agreement; a value between 0.21 and 0.40 indicates a fair agreement; a value between 0.41 and 0.60 indicates a moderate agreement; a value between 0.61 and 0.80 indicates a substantial agreement; and a value between 0.81 and 1.00 indicates an almost perfect agreement.

## Results

### Patient Population Information and Tumor Characteristics

The clinical information of the patients and HNSCC characteristics in this study are summarized in Table 1. The testing cohort included 69 patients (53 males and 16 females). The training cohort included 137 patients (108 males and 29 females). All patients were Chinese, with no patients who were white, black or of other races. Regarding the tumor TNM classifications, only two patients were classified as M1, and the others were classified as M0; therefore, we did not conduct statistical assessments on the M stage. There were no differences between the training and testing cohorts in terms of age, sex, tumor primary location, histological differentiation, TN classification, stage or enhancement types (*p* > 0.05).

**Table 1:** HNSCC patients information and tumor characteristics in the study.

**Information/characteristic**	**Testing cohort**	**Training cohort**	***p*-value**
Age	63.57 ± 12.01 (31–87)	61.18 ± 11.87 (27–86)	0.18
Sex			0.74
Male	53 (76.8%)	108 (78.8%)	
Female	16 (23.2%)	29 (21.2%)	
Tumor primary location			0.45
Oral cavity	35 (50.7%)	71 (51.8%)	
Oropharynx	12 (17.4%)	13 (9.5%)	
Hypoharynx	12 (17.4%)	28 (20.4%)	
Larynx	10 (14.5%)	22 (16.1%)	
Others	0	3 (2.2%)	
Tumor differentiation			0.95
WD	42 (60.9%)	84 (61.3%)	
MD/PD	27 (39.1%)	53 (38.7%)	
T classification			0.64
T1–2	19 (27.5%)	42 (30.7%)	
T3–4	50 (72.5%)	95 (69.3%)	
N classification			0.52
N0	38 (55.1%)	69 (51.1%)	
N+	31 (44.9%)	68 (48.9%)	
Stage			0.79
I–II	14 (20.3%)	30 (21.9%)	
III–IV	55 (79.7%)	107 (78.1%)	
Enhancement types			
Observer 1			0.70
Homogeneous 1	23 (33.3%)	42 (30.7%)	
Heterogeneous 1	46 (66.7%)	95 (69.3%)	
Observer 2			0.23
Homogeneous 2	22 (31.9%)	33 (24.1%)	
Heterogeneous 2	47 (68.1%)	104 (75.9%)	

*Age data are mean ± standard deviation, age range in parentheses, other data are number (percentage). P > 0.05*.

The *p*-value of the kappa statistics analysis was 0.000 (*p* < 0.05), indicating that inter-observer agreement existed. The kappa value was 0.510 [95% CI (confidence interval, CI) 0.379–0.642]. The degree of inter-observer agreement regarding enhancement types was moderate.

**Figure 4:**
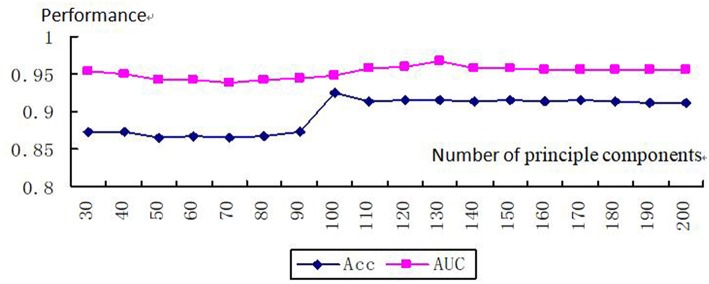
Tuning number of principle components.

After the parameters were finished tuning, a dimension of 130 corresponded to the biggest AUC (AUC = 0.97). Therefore, we obtained a 130-dimensional vector after kernelizing the features of the sample (Figure 4). We built our kernelized model, which used KPCA with a kernelized dimension of 130 as a feature decomposer and random forest classifier, because these parameter values led to the best model performance in terms of AUC.

We obtained the top two features: smoothness and GLCM_t_45_d_1_Con_2. There were significant differences between the WD and MD/PD HNSCC cohorts (*p* < 0.05).

### Performance of the Models

**Figure 5:**
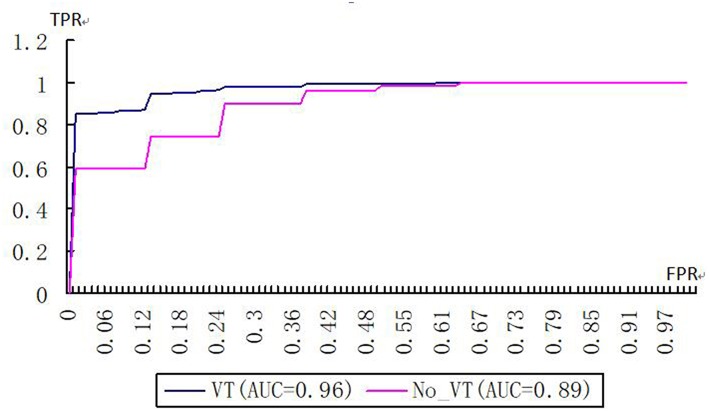
Receiver operating characteristic curves of kernelized models with and without VT selection (FPR false positive rate, TPR true positive rate).

On the basis of VT selection, which eliminated the features with a variance <0.8, the kernelized radiomics model from the CT images achieved the best classification performance. The accuracy, sensitivity, specificity, positive predictive value (PPV), and negative predictive value (NPV) of using the kernelized radiomics models both with and without VT to differentiate WD HNSCC from MD/PD HNSCC are shown in Table 2. ACC, SEN, and NPV in the cohort with VT selection were significantly higher than those without VT selection. The AUCs of the models with VT and without VT are 0.96 and 0.89, respectively (Figure 5). There was significant difference between them (*p* < 0.05).

**Table 2:** The performances of kernelized models with and without VT selection.

	**ACC**	**SEN**	**SPE**	**PPV**	**NPV**	**AUC**
With VT selection	0.92	0.96	0.83	0.94	0.91	0.96
Without VT selection	0.83	0.88	0.70	0.80	0.84	0.89
*p*-*value*	0.002^▴^	0.002^▴^	0.131	0.113	0.000^▴^	0.000^▴^

*ACC, Accuracy; SEN, Sensitivity; SPE, Specificity*.

▴ *p <0.05*.

We used 3-fold validation to split our entire dataset into three parts and recursively used two parts as the training set and one as the testing set. The model was trained on the training set and validated on the testing set, which the model could not learn from. We used ACC, SEN, SPE, PPV, NPV, and AUC to describe the performance of the model, which has the ability to ignore an unbalance between samples with different classes.

**Figure 6:**
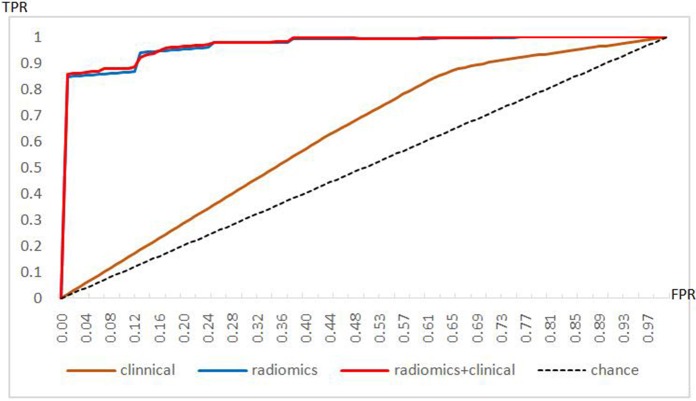
Receiver operating characteristic curves of the performances of three models.

The performances of each model in discriminating tumor grades are summarized in Table 3 and Supplementary Data Sheet 3. The clinical model had lower performance parameters than the radiomics signature and the combined model, and there were significant differences among these models (*p* < 0.05). The combined model had a relatively higher ACC, SEN, and NPV than the radiomics signature, but there were no significant differences between these two models (*p* > *0.05*). The AUCs of the three models are shown in Figure 6. The AUC of the clinical model was much lower than that of the radiomics signature and that of the combined model, which were significant differences (*p* < 0.05). The AUC of the radiomics signature was slightly lower than that of the combined model, but the difference was not significant (*p* > 0.05).

**Table 3:** Discrimination performances of clinical model, radiomics signature features, and the combined model.

**Models**	**ACC**	**SEN**	**SPE**	**PPV**	**NPV**	**AUC**
Clinical^▴^^♀^	0.68	0.87	0.38	0.69	0.68	0.63
Radiomics^*^^▴^	0.92	0.96	0.83	0.94	0.91	0.96
Combined^*^^♀^	0.93	0.97	0.83	0.90	0.92	0.97
^*^	0.72	0.52	1.00	0.97	0.54	0.94
*p* value^▴^	0.00	0.016	0.00	0.00	0.00	0.00
^♀^	0.00	0.003	0.00	0.00	0.00	0.00

* *p > 0.05*,

▴ *p < 0.05*,

♀ *p < 0.05*.

## Discussion

In this study, we combined a RBF KPCA with a random forest classifier for the prediction of HNSCC tumor grade, especially for differentiating WD tumors from MD/PD tumors. A total of 670 features were extracted from each tumor lesion. In total, 130 dimensions were from the PCA based on the highest AUCs at different dimension levels (30–200). These 130 dimensions were used as the inputs for the random forest model. Notably, the application of VT selection to eliminate features with variance <0.8 improved the AUC. We also constructed a clinical and a combined model, and evaluated their performances; the combined model achieved the best performance.

As the solid cancer is spatially and temporally heterogeneous, radiomics is advantageous for non-invasively capturing intra-tumoral heterogeneity from medical imaging (10). Radiomics has been reported for grading brain gliomas and can discriminate high- vs. low-grade gliomas (26–28). Although other modalities such as PET, DWI, histogram analysis of apparent diffusion coefficient (ADC) maps, PWI and DCE-MRI have been used to differentiate the histologic grades of HNSCC (6–8), these multi-parameter imaging methods provide information regarding the composition of HNSCC to reflect metabolism, cellularity, and perfusion. There might exist complex associations among those parameters depending on tumor grade (6). Additionally, intra- and inter-observer variability are important factors in whether these radiology diagnostic tools are independently reliable. In this study, the AUC and ACC of our constructed model were higher than those of PWI (8).

Radiomics is a promising tool for the non-invasive characterization of tumor phenotypes. In our study, we extracted a large number of quantitative features from contrast-enhanced CT images: ROIs were characterized regarding their shape and size features, histogram features, texture features and transformation features. A few radiomics studies have been performed based on MRI to stage and grade HNSCC, and these studies used various methods and obtained some quantitative parameters. Ren et al. (14) also constructed radiomics signatures with the method of least absolute shrinkage and selection operator (LASSO) logistic regression and explored the associations between radiomics signatures and HNSCC stage. The researchers used MRI with contrast-enhanced T1-weighted imaging (CET1WI) and T2-weighted imaging (T2WI) and found that there were three radiomics signatures that were significantly different between stage III-IV and stage I-II in both the testing and training cohorts. Fujima et al. (15) used MRI histograms and a texture analysis of fat-suppressed T2WI to predict the histological grade of HNSCC and found that the relative mean signal and contrast were significantly lower in poorly differentiated SCC than in the well/moderately differentiated SCC. The homogeneity was higher in poorly differentiated SCC than in the well/moderately differentiated SCC. Ahn et al. (16) studied different b values to determine whether histogram analyses of ADC maps can differentiate histologic grades of HNSCC; the researchers found that at a high b value (2,000 s/mm^2^), the mean ADC and kurtosis ratio were significantly different among cohorts of different grades, and the diagnostic accuracies varied among various cohorts.

On the basis of VT selection, which eliminated features with a variance <0.8, the kernelized radiomics model from CT images achieved a good performance. The ACC, SEN, and NPV of the kernelized radiomics models with VT were significantly higher than those of the model without VT. The variance threshold could clearly help improve the performance of the model in grading HNSCC.

Of all 670 features extracted from the portal venous phase contrast-enhanced images, the top two features were smoothness and GLCM_t_45_d_1_Con_2. The smoothness feature concerns the texture of the image, which is either smooth or rough. When the image contains constant gray level intensity values, the texture is smooth. When the intensity levels rapidly vary, the texture is considered rough. In this study, the images of the WD cohort were smoother than those of the MD/PD cohort. We speculated that a WD tumor would resemble normal squamous epithelium, be slightly more keratinized, have slight atypia nuclei, and show less necrosis than a MD/PD tumor; these observations reflect the pathological characteristics of the WD tumor and may relate to smoothness. Regarding the feature GLCM_t_45_d_1_Con_2, GLCM describes the spatial relationship of the pixels and characterizes the image texture by calculating how often pairs of pixels with specific values and spatial relationships occur in an image. HNSCC tumors of different grades have various pathological characteristics, including cellularity, necrosis, vessels, desmoplasia, and inflammatory infiltration, all of which have various pixel values and spatial relationships. Fujima et al. (15) also reported that the contrast and homogeneity parameters of the GLCM texture features based on MRI were significantly different between WD/MD and PD SCC patients. GLCM features may be useful for determining HNSCC grade. Surov et al. (29) reported that ADC histogram parameters represent the proliferation potential and cellularity of HNSCC. In G1/2 and G3 tumors, various ADC parameters correlated with Ki67 expression, cellularity, cell count, and total nucleic area, all of which depend on the tumor grade.

To assess the performance of the radiomics signature for discriminating among HNSCC grades, we additionally constructed two models, a clinical model and a combined model, and compared the performances of these models. Among these three models, the combined model achieved the best performance, although there were no significant differences between the radiomics signature and the combined model. When we incorporated clinical and radiological information into the radiomics signature, the performance of the combined model was not significantly different from that of the radiomics signature, which explains why the radiomics signature also played a predominant role in discriminating between HNSCC grades. A computerized algorithm analysis can make quantitative and qualitative improvements in grading HNSCC tumors with CT images. In prospective radiomics, a signature analysis may serve as a useful, non-invasive tool that is extensively applied in clinical practice.

There were several limitations in our study. First, this was a retrospective and single center study. The study data are limited; multi-center datasets, larger sample data and prospective studies will be needed to validate the performance of our model. Second, the ROIs were subjectively identified by observers according to the most significantly enhanced area inside the tumor on one slice of a CT image. Only 2-dimensional (2D) analysis, rather than 3-dimensional (3D) analysis, was conducted for the radiomics analysis. 3D analyses tend to be more representative of tumor tissue heterogeneity, but a 3D analysis may be more complex and time-consuming. In the future, we will use the automatic segmentation method to define the ROIs. Finally, the methodology used in this study needs to be improved. As machine learning techniques develop, deep learning method has emerged. Convolutional neural network (CNN) is a representative, more advanced method in deep learning. In the future, if the study sample size is enough for deep learning, we will try CNN method for image segmentation and feature extraction. Then the model can be worthy of explaining more.

## Conclusions

In conclusion, in this study, we constructed a radiomics model that could non-invasively discriminate WD HNSCC from MD/PD HNSCC. This radiomics model could be used in precision medicine and improve therapeutic strategies in the clinic. The radiomics model had a better performance with the use of a KPCA, random forest classifier and VT selection and may serve as a potential method for assessing imaging biomarkers for HNSCC patients.

## Data Availability

The datasets generated for this study are available on request to the corresponding author.

## Author Contributions

WW designed and wrote the manuscript. JY designed the imaging analysis method. QW collected and analyzed the data. JL analyzed the data and performed the statistics analysis. SX designed and reviewed the manuscript.

### Conflict of Interest Statement

The authors declare that the research was conducted in the absence of any commercial or financial relationships that could be construed as a potential conflict of interest.
