# Supplementary material for: CT-Based Radiomics Signature for the Preoperative Discrimination Between Head and Neck Squamous Cell Carcinoma Grades
Source: Front Oncol. 2019 Aug 30;9:821. doi: 10.3389/fonc.2019.00821 (PMC6729100; doi:10.3389/fonc.2019.00821)
Supplement: Supplementary file 1 [file Data_Sheet_1.docx]

**TABLE S1** shape and size features were listed in this study

| Shape and size features | | | |
| --- | --- | --- | --- |
| Area | Roundness | Eccentricity | Equivalent diameter |
| Solidity | Minor axis length | Variation | Perimeter |
| Median | Maximum intensity | Kurtosis | Minimum intensity |
| Skewness | Mean intensity | Standard deviation | Major axis length |
| Mean | Orientation |  |  |

**TABLE S2** histogram features were listed in this study

| Histogram features | | | |
| --- | --- | --- | --- |
| Smooth | Root mean square | Energy | Entropy |
| Uniformity | Interquartile range | Percentile histogram 2.5% | Percentile histogram 25% |
| Percentile histogram 50% | Percentile histogram 75% | Percentile histogram 97.5% |  |

**TABLE** **S3** GLCM and GLRLM in texture features were listed in this study

| Texture features | | | | |
| --- | --- | --- | --- | --- |
| GLCM | | GLRLM | | |
| Contrast | Energy | | Run length non-uniformity | Level emphasis |
| Dissimilarity | Difference entropy | | Long run high gray | Low gray level run emphasis |
| Difference variance | Entropy ’SumEntropy’ | | Gray level non-uniformity | Run percentage |
| Sum | Information measure of correlation-1 | | High gray level run emphasis  Short run emphasis | Long run low gray level emphasis |
| Information measure of correlation-2 | GLCM Single Value Decomposition | | Short run high gray level emphasis | Long run emphasis |
| Sum variance | Variance | | Short run low gray level emphasis |  |
| Homogeneity | Angular second moment | |  |  |
| Entropy | Correlation | |  |  |
| Sum average | Means | |  |  |

**TABLE** **S4** Wavelet transform, correlation transform, spatial filtering and local binary patterns (LBP) in transformation features were listed in this study

| \| Transformation features \| \| --- \| | | | | |
| --- | --- | --- | --- | --- | --- |
| Wavelet transform | Correlation transform | | Spatial filtering | LBP |
| Mean | Sum | Mean | Standard Deviation | Histogram of features |
| Variance | Median | Maximum | Skewness |  |
|  | Median | Minimum | Kurtosis |  |
|  | Standard Deviation | Kurtosis | Entropy |  |
